# Glycerol-3-phosphate acyltransferase-1 upregulation by O-GlcNAcylation of Sp1 protects against hypoxia-induced mouse embryonic stem cell apoptosis via mTOR activation

**DOI:** 10.1038/cddis.2015.410

**Published:** 2016-03-24

**Authors:** H J Lee, J M Ryu, Y H Jung, K H Lee, D I Kim, H J Han

**Affiliations:** 1 Department of Veterinary Physiology, College of Veterinary Medicine, Research Institute for Veterinary Science and BK21 Creative Veterinary Research Center, Seoul National University, Seoul, Korea; 2 Department of Veterinary Physiology, College of Veterinary Medicine, Chonnam National University, Gwangju 61186, Korea

## Abstract

Oxygen signaling is critical for stem cell regulation, and oxidative stress-induced stem cell apoptosis decreases the efficiency of stem cell therapy. Hypoxia activates O-linked *β*-N-acetyl glucosaminylation (O-GlcNAcylation) of stem cells, which contributes to regulation of cellular metabolism, as well as cell fate. Our study investigated the role of O-GlcNAcylation via glucosamine in the protection of hypoxia-induced apoptosis of mouse embryonic stem cells (mESCs). Hypoxia increased mESCs apoptosis in a time-dependent manner. Moreover, hypoxia also slightly increased the O-GlcNAc level. Glucosamine treatment further enhanced the O-GlcNAc level and prevented hypoxia-induced mESC apoptosis, which was suppressed by O-GlcNAc transferase inhibitors. In addition, hypoxia regulated several lipid metabolic enzymes, whereas glucosamine increased expression of glycerol-3-phosphate acyltransferase-1 (GPAT1), a lipid metabolic enzyme producing lysophosphatidic acid (LPA). In addition, glucosamine-increased O-GlcNAcylation of Sp1, which subsequently leads to Sp1 nuclear translocation and GPAT1 expression. Silencing of GPAT1 by *gpat1* siRNA transfection reduced glucosamine-mediated anti-apoptosis in mESCs and reduced mammalian target of rapamycin (mTOR) phosphorylation. Indeed, LPA prevented mESCs from undergoing hypoxia-induced apoptosis and increased phosphorylation of mTOR and its substrates (S6K1 and 4EBP1). Moreover, mTOR inactivation by rapamycin (mTOR inhibitor) increased pro-apoptotic proteins expressions and mESC apoptosis. Furthermore, transplantation of non-targeting siRNA and glucosamine-treated mESCs increased cell survival and inhibited flap necrosis in mouse skin flap model. Conversely, silencing of GPAT1 expression reversed those glucosamine effects. In conclusion, enhancing O-GlcNAcylation of Sp1 by glucosamine stimulates GPAT1 expression, which leads to inhibition of hypoxia-induced mESC apoptosis via mTOR activation.

Stem cells in the body are exposed to low oxygen pressure owing to the physiological distribution of vessels.^1^ This hypoxic niche for stem cells is essential to maintain the metabolic characteristics of stem cells.^2^ Thus, describing the oxygen nature of this stem cell niche is important for elucidating stem cell regulation. Oxygen signaling is a major determinant of cell fate-controlling cellular processes. Control of oxygen signaling in stem cells has the potential to regulate embryonic development, cell cultivation, cell reprogramming, and transplantation in regenerative medicine.^1, 3, 4, 5, 6^ There are many reports showing the effects of hypoxia on various kinds of stem cells, and it has been shown that hypoxia has a paradoxical role in stem cell behaviors and cell fate regulation related to stem cell type, ageing, and oxygen concentration.^3, 7, 8, 9^ Studies of mechanisms by which stem cells function under hypoxia, and how they are regulated, have been undertaken. Several investigators recently reported that hypoxia-mediated stem cell metabolic alteration is associated with stem cell function; as a result, interest in the interaction between hypoxia and stem cell metabolism is growing.^10, 11^ However, which metabolic factors are important for stem cell fate under hypoxia have not been elucidated.

O-linked *β*-N-acetyl glucosaminylation (O-GlcNAcylation) is affected by cellular nutrient status and extra-cellular stresses including hypoxia.^12, 13, 14^ A hypoxia-induced glycolytic switch primarily stimulates hexosamine biosynthetic pathway (HBP) flux, which induces O-GlcNAcylation signaling.^15^ O-GlcNAcylation is catalyzed by O-linked N-acetyl glucosamine transferase (OGT) to add N-acetyl glucosamine to the serine or threonine residues of proteins.^16, 17, 18^ O-GlcNAcylation acts as an essential factor for controlling physiological processes including migration, proliferation, and survival in stem cells, and recently it was considered as a potential strategy for use in stem cell therapy.^19, 20, 21^ In addition, as many human metabolic diseases such as diabetes and cancer are attributed to aberrant O-GlcNAcylation, unraveling HBP-mediated O-GlcNAc signaling is important in the development of practical strategies for metabolic diseases treatment. For example, Liu *et al.*^22^ showed that glucosamine-mediated O-GlcNAcylation induced resistance to tissue damage resulting from ischemic injury and provided cardio-protection in an animal model. Furthermore, O-GlcNAcylation interacts with other nutrient metabolic pathways such as lipogenesis, gluconeogenesis, and glycogen synthesis.^12, 23, 24^ Among these metabolic pathways, lipid metabolism is reported to have a central role in controlling stem cell fate.^25, 26^ Collectively, these results suggest that O-GlcNAcylation can be a useful tool for use in cellular metabolic regulation, and identification of an O-GlcNAcylation-regulating potential lipid metabolic factor, which is important for stem cell regulation, may suggest potentially useful metabolic approach in stem cell therapy.

Embryonic stem cells (ESCs) are distinctive in that they have a self-renewal capacity, exhibit pluripotency to enable differentiation into cellular derivatives of three lineages, and may be used as a representative *in vitro* model in the study of early embryo development, pluripotent stem cell physiology, and clinical applications.^27, 28, 29^ Despite the clinical limitation associated with ESCs and the possibility of cancer formation, several studies into the therapeutic effects of ESCs in regenerative medicine have been reported. Indeed, administrations of human or mouse ESCs (mESCs) has induced a paracrine effect and improved damaged cell functions.^30, 31, 32^ However, despite the benefit of ESCs in regenerative medicine, ESC apoptosis remains an impediment to ESC applications using hypoxia.^33, 34, 35^ Thus, researchers are investigating ways to minimize ESC apoptosis and control ESC fate under hypoxia. In this study, we used glucosamine to induce O-GlcNAcylation. Therefore, our study investigated the role of O-GlcNAcylation via glucosamine (GlcN) which is recognized as a HBP activator^36^ in lipid metabolism and in protection of mESC apoptosis under hypoxia.

## Results

### Effect of O-GlcNAcylation on mESC survival under hypoxia

**Figure 1:**
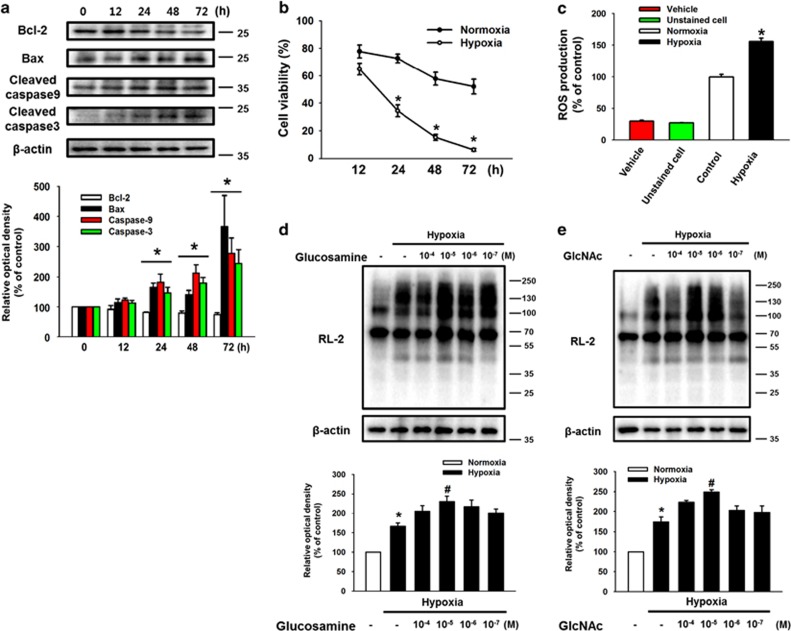
Effects of hypoxia and glucosamine on mouse embryonic stem cells apoptosis. (**a**) Cells were exposed to hypoxic condition for 0–72 h. Bcl-2, Bax, cleaved caspase-9, and cleaved caspase-3 proteins were detected by western blotting. Each result shown is representative of three independent experiments. (**b**) Cell viability was measured directly using a Petroff–Hausser counting chamber (Hausser Scientific, Horsham, PA, USA). Data are reported as mean±S.E.M. of three independent experiments with duplex dishes. *P<0.05 *versus* normoxia control. (**c**) Cells were exposed to hypoxia for 24 h, and then DCF-DA-sensitive cellular ROS was measured by using luminometer. Data are reported as mean±S.E.M. of two independent experiments with triple dishes. *P<0.05 *versus* normoxia control. (**d**, **e**) Cells were treated with various concentrations of glucosamine (GlcN) and GlcNAc (10^−4^ to 10^−7^ M) for 30 min before hypoxia treatment. Glucosamine-pretreated cells were exposed to hypoxia for 24 h; and then, RL-2 and *β*-actin were detected by using western blotting. Each result shown is representative of three independent experiments

**Figure 2:**
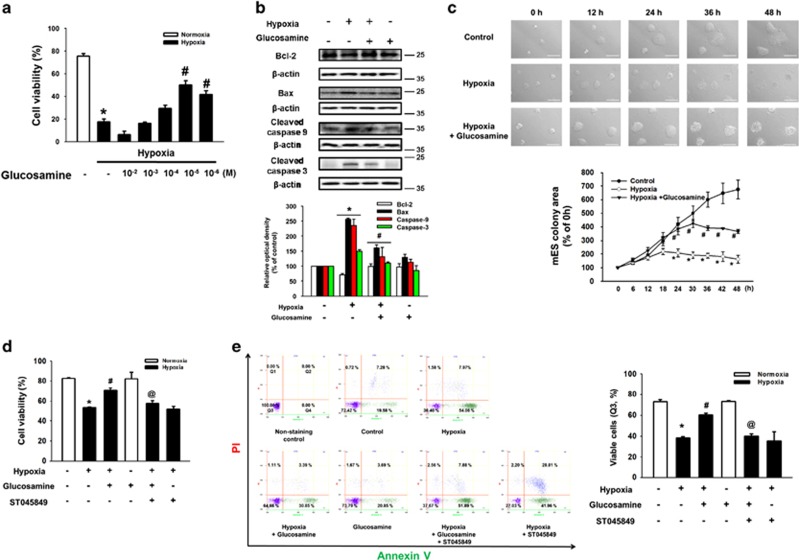
Effect of glucosamine-induced O-GlcNAcylation on mouse embryonic stem cells apoptosis. (**a**) Cells were pretreated with various concentrations of glucosamine (GlcN, 100 mM to 1 *μ*M); and then, exposed to hypoxia for 24 h. Cell viability was detected using by trypan blue exlusion cell viability assay. Data are reported as mean±S.E.M. of two independent experiments with triple dishes. *n*=6, *P<0.05 *versus* normoxia control. (**b**) Cells were pretreated with glucosamine (10 *μ*M) for 30 min before hypoxia treatment. After 24 h of hypoxic incubation, Bcl-2, Bax, cleaved caspase-9, cleaved caspase-3, and *β*-actin proteins were detected by western blotting. Each result shown is representative of three independent experiments. (**c**) mESCs colony size was acquired by using live-cell imaging microscopy system. Cells were exposured to normoxic and hypoxic conditions for 48 h. Data are analyzed by using ImageJ software. Scale bars=100 *μ*m (magnification × 200). Each sample is representative of three independent experiments. *P<0.05 *versus* control, and ^#^*P*<0.05 *versus* hypoxia treatment alone. (**d**) Cells were pretreated ST045849 (20 *μ*M) for 30 min before treatment of glucosamine (10 *μ*M) for 30 min. Subsequently, cells were exposed to hypoxia for 24 h. Cell viability were measured directly using a Petroff–Hausser cell counting chamber. Error bars are reported as mean±S.E.M. of three independent experiments with duplex dishes. *n*=6, *P<0.05 *versus* control, ^#^*P*<0.05 *versus* hypoxia treatment alone, and ^@^*P*<0.05 *versus* hypoxia with glucosamine. (**e**) Cells were immunostained with FITC-conjugated annexin V antibody and PI, and analyzed by flow cytometry. Annexin V-negative-PI-negative cells (Q3) were considered viable, annexin V-negative-PI-positive cells (Q1) were considered necrotic, annexin V-positive-PI-positive cells (Q2) were considered late apoptotic, and annexin V positive-PI-negative cells (Q4) were considered early apoptotic. Data are presented as a mean±S.E.M. of two independent duplex dishes. *P<0.05 *versus* control, ^#^*P*<0.05 *versus* hypoxia treatment alone, and ^@^*P*<0.05 *versus* hypoxia with glucosamine

To examine the effect of hypoxia on mESCs survival, mESCs were incubated under hypoxic condition for various durations (0–72 h). Anti-apoptotic protein Bcl-2 expression level decreased in a time-dependent manner after 12 h of hypoxia. But, hypoxia increased expression levels of Bax, cleaved caspase-9, and cleaved caspase-3 after 12 h of hypoxia (Figure 1a). Viability of hypoxia-treated cells decreased in a time-dependent manner and was significantly lower than that of control cells during 24–72 h of hypoxia treatment (Figure 1b). To investigate the effect of hypoxia on intracellular ROS production of mESCs, we performed DCF-DA staining assays. Intracellular ROS production in mESCs under hypoxia for 24 h increased to 156% of that in the normoxia control (Figure 1c). To confirm the role of glucosamine on O-GlcNAcylation in mESCs, we used RL-2 antibody specific for O-GlcNAc. Hypoxia treatment for 24 h slightly increased total O-GlcNAc level, and the maximum increase in O-GlcNAc level was observed in cells treated with 10 *μ*M of glucosamine and hypoxia (Figure 1d). As shown in Figure 1e, the effect of another O-GlcNAcylation activator GlcNAc was also similar to that of glucosamine. To determine the effect of glucosamine on mESC survival, we examined cell viability after 24 h of hypoxic incubation with various concentrations of the glucosamine (10 mM to 1 *μ*M). Hypoxia significantly decreased cell viability, but viability was recovered by 10 *μ*M of glucosamine treatment (Figure 2a). In addition, hypoxia decreased Bcl-2 expression, and increased Bax, cleaved caspase-9, and cleaved caspase-3 expressions, which were reversed by glucosamine treatment (Figure 2b). Live-cell imaging results showed that the size of mESC colonies with glucosamine under hypoxia was significantly larger than that of the hypoxia controls during 24–48 h incubation (Figure 2c). Next, we investigated whether glucosamine-induced O-GlcNAcylation regulated mESC survival under hypoxia. Addition of the OGT inhibitor ST045849 decreased the previous glucosamine-increased cell viability (Figure 2d). Consistent with this result, the glucosamine treatment reduced annexin V-positive and PI-positive mESCs under hypoxia, which was significantly inhibited by ST045849 addition (Figure 2e).

### Effect of O-GlcNAcylation of Sp1 on GPAT1 expression

**Figure 3:**
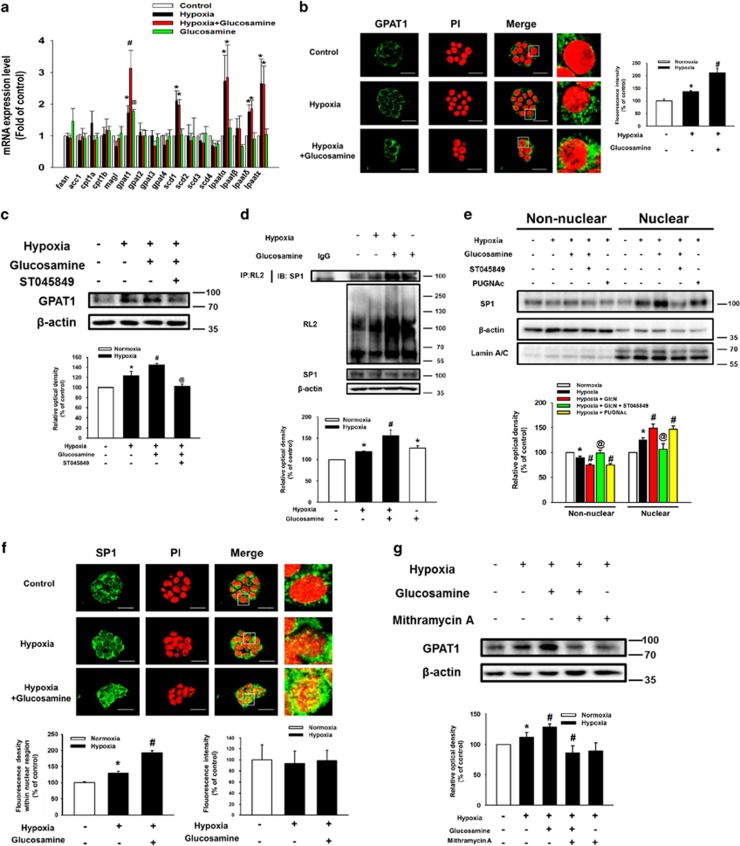
Role of glucosamine-induced O-GlcNAcylation of SP1 in GPAT1 expression. Cells were pretreated with glucosamine (10 *μ*M) for 30 min before hypoxia treatment; and then, cells were exposed to hypoxia for 24 h. (**a**) Total mRNA from mESCs was reverse-transcribed and *fasn, acc1, cpt1a, cpt1b, magl, gpat1, gpat2, gpat3, gpat4, scd1, scd2, scd3, scd4, lpaatα, lpaatβ, lpaatδ, lpaatɛ, and β-actin* mRNA amplified by PCR. The mRNA expression levels were measured by using real-time PCR. Each mRNA expression was normalized by *β-actin* mRNA expression level. Data are presented as a mean±S.E.M. of three independent duplex dishes. *P<0.05 *versus* control, ^#^*P*<0.05 *versus* hypoxia treatment alone, and ^@^*P*<0.05 *versus* hypoxia with glucosamine. (**b**) Cells were immune-stained with GPAT1 antibody and PI. Fluorescence intensity (ImageJ arbitrary units) of GPAT1 was analyzed by using ImageJ software (developed by Wayne Rasband, National Institutes of Health, Bethesda, MD, USA; http://rsb.info.nih.gov/ij/). The result images are representative of three independent experiments. Data are presented as a mean±S.E.M. of three independent experiments. *P<0.05 *versus* control, ^#^*P*<0.05 *versus* hypoxia treatment alone. Scale bars=25 *μ*m (magnification × 800). (**c**) Cells were pretreated with ST045849 (20 *μ*M) for 30 min before glucosamine treatment (10 *μ*M) for 30 min, and then cells were exposed to hypoxia for 24 h. GPAT1 protein expression was measured by using western blotting. Each result shown is representative of three independent experiments. *P<0.05 *versus* control, ^#^*P*<0.05 *versus* hypoxia treatment alone, and ^@^*P*<0.05 *versus* hypoxia with glucosamine. (**d**) O-GlcNAcylation of Sp1 was measured with an immunoprecipitation assay as described in the Materials and methods section. RL-2, Sp1, and *β*-actin protein expressions were detected by western blotting. Each result shown is representative of three independent experiments. (**e**) Cells were pretreated with ST045849 (20 *μ*M) or PUGNAc (10 *μ*M) before glucosamine (10 *μ*M) treatment, and then cells were exposed to hypoxia for 24 h. Collected cells were fractionized into non-nuclear and nuclear fractions. Sp1, *β*-actin, and lamin A/C were detected by western blotting. Each result shown is representative of three independent experiments. *P<0.05 *versus* control, ^#^*P*<0.05 *versus* hypoxia treatment alone, and ^@^*P*<0.05 *versus* hypoxia with glucosamine. (**f**) GPAT1 was detected by immune-staining with GPAT1 antibody. Fluorescence intensity of total GPAT1 and GPAT1 within nuclear region were analyzed by using ImageJ software. Error bars are presented as a mean±S.E.M. of three independent experiments. *P<0.05 *versus* control, ^#^*P*<0.05 *versus* hypoxia treatment alone. Scale bars=25 *μ*m (magnification × 800). (**g**) Cells were pretreated mithramycin A (1 *μ*M) for 30 min before glucosamine (10 *μ*M) treatment for 30 min. Subsequently, cells were exposed to hypoxia for 24 h. Total protein was extracted, and blotted with GPAT1. Each result shown is representative of three independent experiments

To determine the effect of hypoxia and glucosamine on lipid metabolic enzyme expressions in mESCs, we assessed mRNA expression levels of fatty acid synthase (*fasn*), acetyl-coenzyme A carboxylase 1 *(acc1*), carnitine palmitoyltransferase 1a (*cpt1a*)*, cpt1b,* monoacylglycerol lipase (*magl*), *gpat1, gpat2, gpat3, gpat4,* stearoyl-CoA desaturase 1 (*scd1*)*, scd2, scd3, scd4,* lysophosphatidic acid acyltransferase-*α* *(lpaatα*)*, lpaatβ, lpaatδ,* and *lpaatɛ.* Hypoxia increased the mRNA expression levels of *gpat1, scd1, lpaatα, lpaatδ,* and *lpaatɛ*, whereas glucosamine treatment increased only *gpat1* mRNA expression (Figure 3a). Immunofluorescence staining results showed a 210% increase in the fluorescence intensity of GPAT1 in the glucosamine and hypoxia-treated mESCs and a 137% increase in the fluorescence intensity of GPAT1 in hypoxia-treated mESCs (Figure 3b). Furthermore, glucosamine-induced GPAT1 expression was inhibited by ST045849 pretreatment (Figure 3c). However, tunicamycin, a N-linked glycosylation inhibitor, did not affect glucosamine-induced GPAT1 expression of mESCs under hypoxia (Supplementary Figure S1). As shown in Figures 3d and e, hypoxia and glucosamine stimulated O-GlcNAcylation and nuclear translocation of Sp1. Nuclear translocation of Sp1 was regulated by the addition of ST045849 and the OGA inhibitor PUGNAc. After 24 h of hypoxia and hypoxia with glucosamine treatments, fluorescence intensity of Sp1 in the PI-stained region increased to 129% and 193%, respectively. However, total fluorescence intensity of Sp1 was not affected by hypoxia and glucosamine treatment (Figure 3f). In addition, pretreatment with the Sp1 inhibitor mithramycin A (1 *μ*M) suppressed glucosamine-induced GPAT1 expression (Figure 3g). However, the inhibition of SREBP1 activity by fatostatin pretreatment did not affect glucosamine-induced GPAT1 expression (Supplementary Figure S2). On the basis of these results, we suggest that O-GlcNAcylation has a critical role in glucosamine-induced Sp1 nuclear translocation, which is followed by regulation of GPAT1 expression in mESCs under hypoxia.

### Role of GPAT1 in mTOR activation and mESC apoptosis under hypoxia

**Figure 4:**
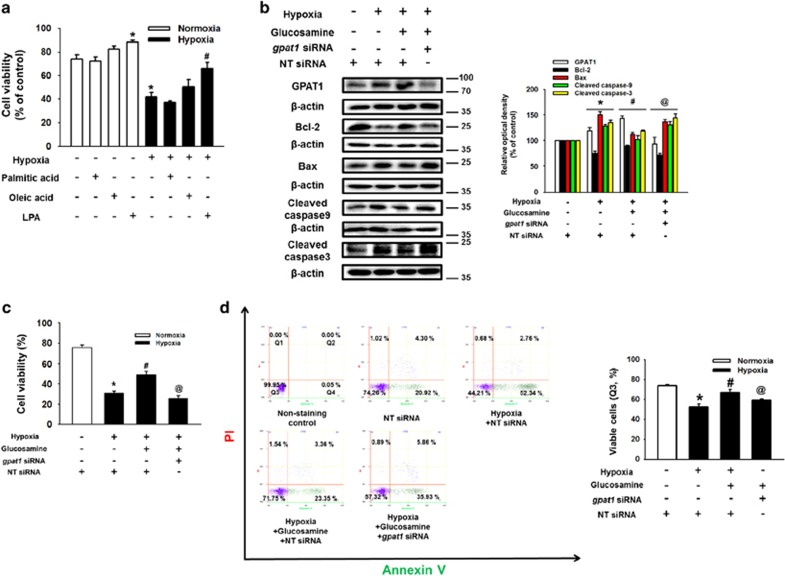
Role of GPAT1 in mESCs apoptosis under hypoxia. (**a**) Cells were pretreated with palmitic acid (10 *μ*M), oleic acid (10 *μ*M), and LPA (0.1 *μ*M) before hypoxia treatment for 24 h. Cell viability was measured with trypan blue exclusion assay. Error bars are showed as a mean±S.E.M. of three independent experiments with duplex dishes. *P<0.05 *versus* control, ^#^*P*<0.05 *versus* hypoxia treatment alone. (**b**) Cells were transfected with *gpat1* and non-targeting (NT) siRNA for 24 h before glucosamine (10 *μ*M) treatment for 30 min, and then cells were exposed to hypoxia for 24 h. Total lysates were blotted with GPAT1, Bcl-2, Bax, caspase-9, caspase-3, and *β*-actin. The NT siRNA was used as a negative control. Each blot was representative image of three independent experiments. *P<0.05 *versus* control, ^#^*P*<0.05 *versus* hypoxia treatment alone, and ^@^*P*<0.05 *versus* hypoxia with glucosamine. (**c**) Cell viability was measured directly by trypan blue exclusion assay using counting chamber. Data are presented as a mean±S.E.M. of three independent experiments with duplex dishes. *P<0.05 *versus* control, ^#^*P*<0.05 *versus* hypoxia treatment alone, and ^@^*P*<0.05 *versus* hypoxia with glucosamine. (**d**) Viable cells were measured by using annexin V/PI flow cytometry analysis. Annexin V-negative-PI-negative cells (Q3) were considered viable, annexin V-negative-PI-positive cells (Q1) were considered necrotic, annexin V-positive-PI-positive cells (Q2) were considered late apoptotic, and annexin V-positive-PI-negative cells (Q4) were considered early apoptotic. Data are presented as a mean±S.E.M. of two independent duplex dishes. *P<0.05 *versus* control, ^#^*P*<0.05 *versus* hypoxia treatment alone, and ^@^*P*<0.05 *versus* hypoxia with glucosamine

**Figure 5:**
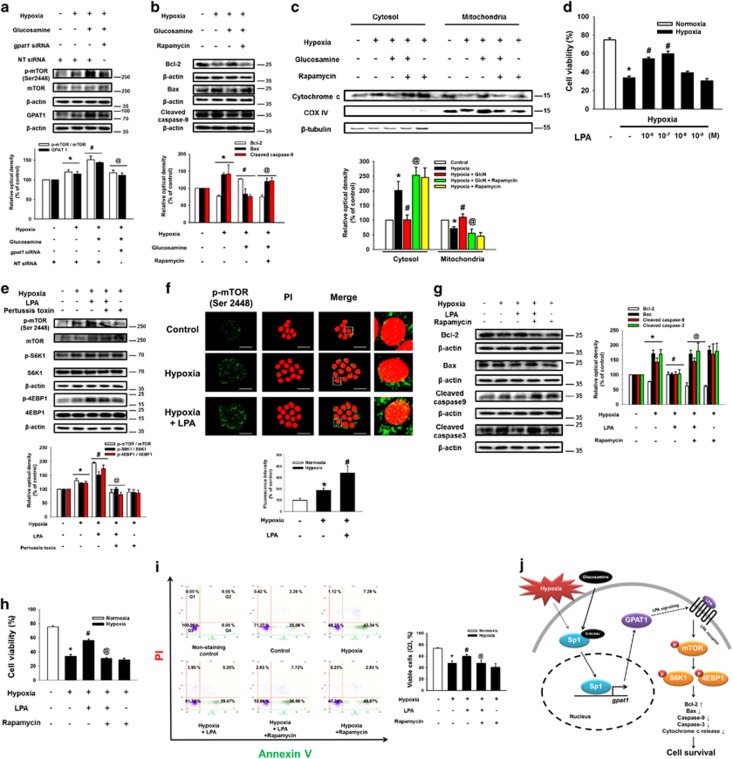
Involvement of mTOR in GPAT1-induced mESCs anti-apoptosis under hypoxia. (**a**) Cells were transfected with *gpat1* and NT siRNA for 24 h before glucosamine (10 *μ*M) for 30 min. Subsequently, cells were exposed to hypoxia treatment for 24 h. Collected samples are lysed, and p-mTOR, GPAT1, and *β*-actin protein expressions were measured by using western blotting. Each result shown is representative of three independent experiments. *P<0.05 *versus* control, ^#^*P*<0.05 *versus* hypoxia treatment alone, and ^@^*P*<0.05 *versus* hypoxia with glucosamine. (**b**) Cells were pretreated with rapamycin (10 nM) before glucosamine (10 *μ*M) treatment; and then, cells were exposed to hypoxia for 24 h. Total proteins were extracted, and blotted with Bcl-2, Bax, cleaved caspase-9, and *β*-actin. Each result shown is representative of three independent experiments. (**c**) Cells were pretreated with glucosamine and/or rapamycin (10 nM) for 30 min before hypoxia treatment, and cytochrome c, COX IV, and *β*-tubulin in the cytosolic and mitochondrial fraction were detected by western blot. (**d**) Cells were pretreated with various concentrations of LPA (10^−^^6^ M–10^−^^9^ M) before hypoxia treatment for 24 h. Cell viability was measured by trypan blue exclusion assay. Error bars are presented as a mean±S.E.M. of three independent duplex dishes. *P<0.05 *versus* control, ^#^*P*<0.05 *versus* hypoxia treatment alone. (**e**) Cells were pretreated with pertussis toxin (100 ng/ml) for 30 min before LPA treatment (0.1 *μ*M) for 30 min. Subsequently, cells were exposed to hypoxia treatment for 24 h. Total proteins were extracted and blotted with p-mTOR, mTOR, p-S6K1, S6K1, p-4EBP1, 4EBP1, and *β*-actin. Each result shown is representative of three independent experiments. *P<0.05 *versus* control, ^#^*P*<0.05 *versus* hypoxia treatment alone, and ^@^*P*<0.05 *versus* hypoxia with LPA. (**f**) p-mTOR was immunostained with p-mTOR antibody, and counter-stained with PI. Fluorescence images were acquired by using confocal microscopy. Fluorescence intensity of p-mTOR was quantified by using ImageJ software. Data are presented as a mean±S.E.M. of three independent experiments. *P<0.05 *versus* control, ^#^*P*<0.05 *versus* hypoxia treatment alone. (**g**) Cells were pretreated with rapamycin (10 nM) before LPA (0.1 *μ*M) treatment; and then, cells were exposed to hypoxia for 24 h. Total proteins were extracted, and blotted with Bcl-2, Bax, cleaved caspase-9, cleaved caspase-3, and *β*-actin. Each result shown is representative of three independent experiments. (**h**) Cell viability was measured by using cell counter. Data are presented as a mean±S.E.M. of three independent duplex dishes. *P<0.05 *versus* control, ^#^*P*<0.05 *versus* hypoxia treatment alone, and ^@^*P*<0.05 *versus* hypoxia with LPA. (**i**) Viable cells were measured by using annexin V/PI flow cytometry analysis. Annexin V-negative-PI-negative cells (Q3) were considered viable, annexin V-negative-PI-positive cells (Q1) were considered necrotic, annexin V-positive-PI-positive cells (Q2) were considered late apoptotic, and annexin V-positive-PI-negative cells (Q4) were considered early apoptotic. Data are presented as a mean±S.E.M. of two independent duplex dishes. *P<0.05 *versus* control, ^#^*P*<0.05 *versus* hypoxia treatment alone, and ^@^*P*<0.05 *versus* hypoxia with LPA. *P<0.05 *versus* control, ^#^indicates *P*<0.05 *versus* hypoxia treatment alone, and ^@^*P*<0.05 *versus* hypoxia with LPA. The proposed model for signaling pathways involved in glucosamine-induced mESCs survival under hypoxia (**j**)

Next, to determine the effect of lipid metabolic enzymes' metabolites on mESC survival under hypoxia, we performed cell viability assays after mESC pretreatment with palmitic acid, oleic acid, and LPA. As shown in Figure 4a, the survival effect of LPA was stronger than that of other substrates in mESCs under normoxia or hypoxia. Subsequently, we treated mESCs with glucosamine and *gpat1*-specific siRNA to assess the role of GPAT1 on mESC survival under hypoxia. Silencing of GPAT1 expression decreased Bcl-2 expression, but increased Bax, cleaved caspase-9, and cleaved caspase-3 expressions (Figure 4b). We performed trypan blue exclusion cell viability assays and annexin V/PI FACS analysis to elucidate further the role of GPAT1. The cell viability of *gpat1* siRNA with glucosamine pretreatment was similar to that of hypoxia-treated mESCs (Figures 4c and d). In addition, hypoxia and glucosamine-induced GPAT1 did not affect undifferentiation markers Oct3/4 and nanog expressions (Supplementary Figure S3). These results indicate that glucosamine-induced GPAT1 upregulation in hypoxia significantly affects mESC survival. Subsequently, we investigated the detailed signaling pathway by which GPAT1 induces mESC survival under hypoxia. As shown in Figure 5a, glucosamine treatment phosphorylated mTOR, which was blocked by *gpat1* siRNA transfection. We confirmed that glucosamine treatment increased Bcl-2 expression, and decreased Bax, cleaved caspase-9 expressions and cytochrome c release, which were reversed by 10 nM of mTOR inhibitor rapamycin pretreatment (Figures 5b and c). In addition, glucosamine treatment increased [^3^H]-thymidine incorporation level of mESCs under hypoxia, which was blocked by rapamycin addition (Supplementary Figure S4). Next, we treated LPA to determine the role of GPAT1 metabolite in mESC apoptosis under hypoxia. We examined cell viability after 24 h of hypoxic incubation with various concentrations of LPA (1 *μ*M to 1 nM). The 1 *μ*M and 0.1 *μ*M LPA pretreatments significantly increased the cell viability of mESCs under hypoxia (Figure 5d). In addition, hypoxia and hypoxia with 1 *μ*M of LPA pretreatments increased phosphorylation of mTOR and mTOR substrates (S6K1 and 4EBP1). The LPA-induced phosphorylation of mTOR and mTOR substrates were suppressed by pretreatment with the LPA receptor inhibitor Ptx (Figure 5e). After hypoxia and hypoxia with LPA treatment, the fluorescence intensity of p-mTOR in mESC colonies increased to 187% and 342%, respectively (Figure 5f). To elucidate the role of mTOR in mESC survival under hypoxia, we assessed expression levels in apoptosis-related proteins. Treatment with LPA increased Bcl-2 expression and decreased Bax, cleaved caspase-9, and cleaved caspase-3 expressions, which were reversed by rapamycin addition (Figure 5g). Cell viability assay and annexin V/PI FACS analysis results showed that rapamycin treatment reduced LPA-induced mESC survival (Figures 5h and i). In addition, we performed several experiments to identify the role of NF-κB in mESC apoptosis under hypoxia. We found that LPA treatment increased phosphorylation (Ser 536) of NF-*κ*B, the activation marker (Supplementary Figure S5). However, NF-*κ*B inhibitor SN-50 did not affect LPA-induced survival effect on mESCs under hypoxia (Supplementary Figure S6). On the basis of these results, we suggest that LPA activates the mTOR pathway, resulting in mESC survival under hypoxia.

### Role of GPAT1 on mESC survival and skin flap survival

**Figure 6:**
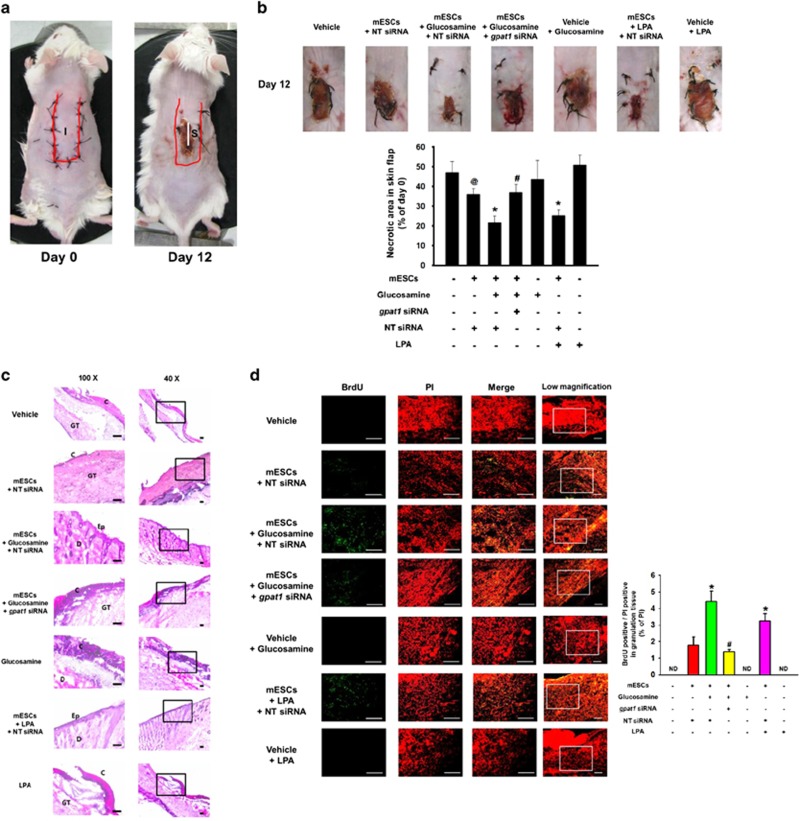
Role of GPAT1 in mESCs survival in the mouse skin flap model. Mouse skin flap surgery with BrdU-harbored mESCs transplantation was performed as described in Materials and methods section. (**a**) NT siRNA-transfected cells are injected in the center region (I) of the flap. At post-injection day 12, all tissue samples (S) including injection site in are excised and collected. (**b**) Representative gross images of skin flap were obtained at day 12 after flap surgery. Necrotic area in skin flap was analyzed by using ImageJ software. Error bars indicate a mean±S.E.M. *n*=5. ^@^*P*<0.05 *versus* vehicle group, *P<0.05 *versus* mESCs group, and ^#^*P*<0.05 *versus* mESCs with glucosamine group. (**c**) Tissue samples were stained with hematoxylin and eosin. Histological images shown in result are representative. Scale bars, 100 *μ*m (magnification, × 40 and × 100). (**d**) BrdU was immunostained with BrdU specific antibody and PI for nuclear counting, and samples were visualized by using confocal microscopy. BrdU and PI-stained cells were analyzed by using MetaMorph software. Scale bars, 200 *μ*m (magnification, × 100 and × 200). *P<0.05 *versus* mESCs group, and ^#^*P*<0.05 *versus* mESCs with glucosamine group. C, crust; Ep, epidermis; D, dermis; GT, granulated tissue; ND, not detected

We performed mouse skin flap surgery to test the protective roles of glucosamine and GPAT1 on transplanted mESC survival. Twelve days after surgery, an extended necrotic area in the central and distal part of the flap appeared in the vehicle, glucosamine, and LPA alone control groups. The necrotic area of the NT siRNA-transfected mESCs alone group was smaller than that of vehicle group. The necrotic region in the skin flap was reduced to a significantly greater extent in NT siRNA-transfected mESCs with either glucosamine or LPA treatment than that in the NT siRNA-transfected mESCs alone group. However, the necrotic area of the *gpat1* siRNA-transfected mESCs with glucosamine treatment group was larger than that of the NT siRNA-transfected mESCs with glucosamine treatment group (Figure 6b). Histological evaluation, via H&E staining, showed an intact epithelial layer in the NT siRNA-transfected mESCs with glucosamine or LPA treatments, which indicates that mESCs with glucosamine treatment can stimulate skin flap survival under ischemic conditions (Figure 6c). Immunohistochemistry results showed that glucosamine and LPA significantly increased the number of BrdU-positive mESCs in the skin flap. The number of *gpat1* siRNA-transfected mESCs in the glucosamine treatment group was similar to that in the NT siRNA-transfected mESCs alone group (Figure 6d).

## Discussion

The results of the present investigation demonstrate the role of GPAT1 expression via augmented O-GlcNAcylation in mESC survival under hypoxia. Although the effect of hypoxia on stem cell survival is not fully elucidated,^3, 4^ we observed that increasing the exposure of mESC to hypoxia resulted in duration-dependent apoptosis. Mishra *et al.*^37^ reported that an increase in the Bax/ Bcl-2 ratio is essential for caspase-9 mediated mitochondrial apoptosis under hypoxia. In this study, hypoxia and glucosamine treatments increased O-GlcNAc level, and glucosamine-augmented O-GlcNAcylation suppressed hypoxia-induced mESC apoptosis. In support of our results, there are several reports that glucosamine activates HBP flux and has a protective role via O-GlcNAcylation in other types of cells including cardiomyocytes and retinocytes.^36, 38, 39^ Moreover, our previous study established that glucosamine induces OGT expression, which is followed by an increase in O-GlcNAc levels in mESCs.^40^ However, a high dose (>1 mM) of glucosamine did not induce the mESC protective effect. There are several reports showing that a high level of glucosamine-induced HBP activation generates excess ROS, leading to apoptosis.^41, 42^ In addition, there are several reports suggesting that O-GlcNAc signaling contributes to undifferentiation and self-renewal.^43, 44^ Furthermore, several reports suggest that hypoxia-induced O-GlcNAc signaling regulates metabolic alteration as well as survival responses against noxious stimuli in stem cells.^45, 46, 47^ These results indicate the need to clarify the interaction of O-GlcNAc signaling-mediated cell survival with metabolic alteration. Based upon current and past results, we suggest that O-GlcNAcylation is a key factor in maintaining of stem cell populations *in vivo*.

There are several recent reports on the interaction between lipid metabolic alteration by hypoxia and stem cell regulation. Indeed, several researchers have reported that hypoxia modulates lipid metabolic processes including fatty acid synthesis and *β*-oxidation.^48, 49^ Our present study shows that hypoxia increased levels of metabolic enzymes that contribute to fatty acid and bio-lipid metabolite production and phosphatidic acid signaling. These lipid metabolites have been reported to have the ability to control stem cell functions such as migration, proliferation, and survival.^50, 51, 52^ In addition, alteration of lipid metabolic enzymes profiles by hypoxia appears stem cell type-specific. Our previous paper reported that hypoxia predominantly increased FASN in human mesenchymal stem cells (hMSCs), which regulates hMSC proliferation and migration.^11^ Ben-David *et al.*^50^ reported that SCD1 inhibition eliminated undifferentiated human ESCs selectively, but our previous study showed that SCD1 inhibition did not affect hMSC proliferation. Taken together, these results indicate a difference in lipid sensitivity between stem cell types. In particular, it is noteworthy that glucosamine up-regulates GPAT1 expression through O-GlcNAcylation, which has an important role in mESC survival under hypoxia. GPAT1 is a lipid metabolic enzyme localized at the mitochondrial outer membrane and is a major form of four GPAT isotypes.^53, 54^ GPAT1 metabolizes glycerol-3-phosphate with long chain fatty acyl-CoA to form LPA.^55^ Although no reports have demonstrated a role of GPAT1 in stem cells, several investigators have reported that GPAT1 is a factor potentially controlling various cellular processes such as lipid metabolism, mitochondrial dynamics, apoptosis, and proliferation in other cell types.^56, 57, 58^ Further investigation into GPAT1's effect and the detailed mechanism for that effect in stem cells will provide information important for stem cell regulation. As Sp1 is emerging as a potential therapeutic target in metabolic diseases, interest in its effect on metabolic and nutritional regulation is increasing.^59, 60, 61^ Furthermore, Sp1 is a transcription factor binding to the human GPAT1 promoter and regulating gene expression.^55, 62^ In addition, there are several reports suggesting that a HBP flux-mediated protective effect is induced via O-GlcNAcylation of specific proteins.^63, 64^ Moreover, O-GlcNAcylation of Sp1 is important for its subcellular localization, stabilization, and transcriptional regulation.^60, 65, 66^ Taken together, these findings indicate that Sp1 regulation by O-GlcNAcylation can be a practical approach to metabolic regulation of stem cell fate.

Furthermore, our results demonstrate that O-GlcNAcylation-mediated GPAT1 expression significantly suppresses hypoxia-induced mESC mitochondrial apoptosis and increases proliferation via mTOR activation, which may suggest that glucosamine-induced resistance against hypoxia is attributed to GPAT1 signaling-mediated mTOR activation. The GPAT1 metabolite LPA has been reported as a potential bioactive lipid molecule that can modulate stem cell function.^67, 68, 69, 70^ You *et al.*^71^ reported that LPA activates the mTOR pathway via an LPA receptor-mediated ERK activation pathway. In addition, there are reports that mTOR is a nutrient-sensing molecule and can regulate stem cell survival.^72, 73, 74^ A previous study reported that AKT/mTOR pathway activation increased the expression of anti-apoptotic proteins Bcl-2 and Bcl-xL, resulting in cell survival and growth. This result suggests that there may be an interaction between GPAT1 signaling-mediated mTOR activation and apoptosis-related protein expression.^75^ In this study we demonstrate that glucosamine-induced GPAT1 expression increased mESC survival after transplantation; moreover, it prevented ischemia-induced flap necrosis *in vivo* in an animal model. The effects of glucosamine and GPAT1 on transplanted stem cell survival demonstrated in this study may have clinical implications for stem cell-based treatments. To our knowledge, this is the first detailed identification of signaling pathways that allow glucosamine-induced O-GlcNAcylation to control lipid metabolic alteration and provide cytoprotection against hypoxia in stem cells (Figure 5j); thus, suggesting that lipid metabolic regulation via O-GlcNAcylation could be a novel strategy in stem cell therapy. Further investigation into the identification of key metabolic pathways that can regulate stem cell fate and function may hold additional promise for various stem cell applications. In conclusion, our results show that O-GlcNAcylation of SP1-induced GPAT1 expression is critical for mESC survival via mTOR activation under hypoxia.

## Materials and Methods

### Materials

Cells from a mESC line (ES-E14TG2a) were provided by the American Type Culture Collection (Manassas, VA, USA). Fetal bovine serum was purchased from HyClone (Logan, UT, USA). The Bcl-2, Bax, caspase-3, caspase-9, Sp1, cytochrome c, COX IV, *β*–tubulin, Oct3/4, nanog, *β*-actin, and Lamin A/C antibodies were acquired from Santa Cruz Biotechnology (Dallas, TX, USA). The RL-2, Glycerol-3-phosphate acyltransferase-1 (GPAT1), and ALG10 antibodies were obtained from abcam (Cambridge, MA, USA). Mammalian target of rapamycin (mTOR), p-mTOR (Ser 2448), S6K1, p-S6K1 (Thr 389), 4EBP1, and p-4EBP1 (Thr 37/46) antibodies were purchased from Cell Signaling Technology (Beverly, MA, USA). Horseradish peroxidase (HRP)-conjugated rabbit anti-mouse and goat anti-rabbit secondary antibodies were acquired from Thermo Fisher (Waltham, MA, USA), whereas HRP-conjugated rabbit anti-goat secondary antibody was obtained from Santa Cruz Biotechnology. Rapamycin, D-glucosamine, PUGNAc, N-acetyl D-glucosamine (GlcNAc), tunicamycin, lysophosphatidic acid (LPA), palmitic acid, oleic acid, mithramycin A, pertussis toxin (Ptx), SN-50, and bromodeoxyuridine (BrdU) were purchased from Sigma-Aldrich (St. Louis, MO, USA). The OGT inhibitor ST045849 was obtained from Timtec (Newark, DE, USA). Small interfering RNAs (siRNA) for *gpat1* and non-targeting (NT) siRNA were acquired from Dharmacon (Lafayette, CO, USA). Propidium iodide (PI) and Alexa fluor 488-conjugated secondary antibodies were purchased from Life technologies (Gaithersburg, MD, USA). All experiments were performed with 6–10 passages of the tested cells.

### mESC culture

The mESCs were cultured with 15% FBS, 1% antibiotic mixture, 5 ng/ml mouse leukemia inhibitory factor (LIF), and high glucose Dulbeco's essential medium (DMEM; Gibco, Grand Island, NY, USA). The cells were grown on six-well plates or in 35 or 60 mm culture dishes in an incubator maintained at 37 °C with 5% CO_2_. After 48 h of cell culture, cells were washed twice with phosphate buffered solution (PBS) and placed in DMEM-supplemented culture medium with 5% serum replacement, 1% antibiotics, and 5 ng/ml LIF (SR-medium). After cells were incubated 24 h for mESC synchronization, cells were washed twice with PBS and placed in SR-medium with agents.

### Hypoxia treatment

For hypoxia treatment, a modular incubation chamber (Billups-Rothenberg, Del Mar, CA, USA) was used. The hypoxic gas included 2.2% O_2_, 5.5% CO_2_, and 92.3% N_2_. Synchronized mESCs were placed in the incubation chamber, and the chamber was purged with the hypoxic gas at a rate of 5 l/min for 30 min. The chamber was then sealed and placed in a conventional incubator kept at 37 °C.

### Mouse skin flap model

All procedures using animals followed the National Institutes of Health Guidelines for the Humane Treatment of Animals and were performed under Institutional Animal Care and Use Committee–approved protocols at Seoul National University (SNU-150508-4). Mice were housed in a standard animal facility maintained on a 12 h light/dark cycle and within a room temperature range of 20–25 °C. Eight-week-old male Institute for Cancer Research mice were anesthetized with 2% isoflurane in oxygen/nitrous oxide mixtures. A previously described skin flap model procedure was performed.^76, 77, 78^ Experimental animals were divided into seven groups: vehicle-injected wild-type mice (group 1, *n*=6); mESC transplantation mice that received mESC pretreated with either NT siRNA alone (group 2, *n*=6) or NT siRNA and 10 *μ*M glucosamine (group 3, *n*=6); mice that received mESC pretreated with *gpat1* siRNA and 10 *μ*M glucosamine (group 4, *n*=6); mice that received 10 *μ*M glucosamine with vehicle (group 5, *n*=6); mice that received mESC pretreated with 0.1 *μ*M LPA (group 6, *n*=6); and mice that received 0.1 *μ*M LPA with vehicle (group 7, *n*=6). After shaving off the hair, a 4 cm × 1 cm skin flap template on the dorsal surface of the mouse was traced by using a surgical marker. The surgical procedure for flap creation was performed under aseptic conditions. A full-thickness skin flap was elevated for 30 min. For treatment, 100 *μ*l of a PBS and matrigel (BD Biosciences, Franklin Lakes, NJ, USA) mixture containing vehicle or mESCs (*n*=1 × 10^6^) with either glucosamine or LPA was injected into the dermis at the center of the skin flap. The flap was then sutured back to its original position by a simple interrupted suturing technique using 3-0 silk non-absorbable sutures (Figure 6a). Transplanted mESCs were pretreated with BrdU (2 *μ*M). All flap images were acquired at the same distance from the subject (30 cm) with a digital camera system (D50; Nikon, Tokyo, Japan). At post-injection day 12, all mice were killed and 1.5 cm × 0.5 cm tissue samples in the central part of the skin flap were excised and collected (Figure 6a). Collected tissues were embedded in O.C.T compound (Sakura Finetek, Torrance, CA, USA) and frozen. Embedded samples were sliced (10-*μ*m thick), and underwent hematoxylin and eosin (H&E) and immunohistochemical (IHC) staining. Sliced samples for IHC analysis were immunostained with BrdU and PI. Immunostained cells were visualized by using fluorescence microscopy (Olympus, Tokyo, Japan), and the images were analyzed by using MetaMorph software (Universal Imaging, West Chester, PA, USA). Visual assessment of skin flap necrotic areas was performed by using ImageJ software (developed by Wayne Rasband, National Institutes of Health, Bethesda, MD, USA; http://rsb.info.nih.gov/ij/). Dark areas with scabs in a skin flap were considered necrotic. To determine the portion of the skin flap that was necrotic, we used the formula: Necrotic area in skin flap=necrotic area of flap area/area of total flap × 100.

### Western blot analysis

After normoxic or hypoxic incubation, cells were collected by using a cell-collecting scraper. Collected samples were washed twice with ice-cold PBS prior to incubation in RIPA lysis buffer (Thermo Fisher) containing proteinase and phosphatase inhibitor (Thermo Fisher) for 30 min on ice. Next, the lysates were centrifugated for clearance (15 000 r.p.m. at 4 °C for 30 min). The protein concentration in the lysate was determined by using a bicinchoninic acid assay kit (Bio-Rad, Hercules, CA, USA). Obtained protein (10 *μ*g) was then loaded in 10% SDS-polyacryl-amide gel for electrophoresis and transferred to a polyvinylidene fluoride (PVDF) membrane. The protein-transferred membrane was washed with tris-buffered saline containing a 0.1% Tween-20 (TBST) solution (10 mM Tris-HCl (pH 7.6), 150 mM NaCl, and 0.1% Tween-20) and blocked with 5% skim milk or 5% bovine serum albumin for 15 min. The membranes were then washed with TBST solution for 30 min and incubated with primary antibody (1:1000 dilution) overnight at 4 °C. The membranes were washed again and then incubated with HRP-conjugated secondary antibody (1:10 000 dilution) for 6 h at 4 °C. The western blotting bands were detected by using enhanced chemiluminescence (Bio-Rad). Densitometric analysis of western blotting bands was quantified by using ImageJ software.

### Nuclear and Non-nuclear fraction preparation

Harvested samples were suspended in buffer A solution (137 mM NaCl, 8.1 mM Na_2_HPO_4_, 2.7 mM KCl, 1.5 mM KH_2_PO_4_, 2.5 mM EDTA, 1 mM dithiothreitol, 0.1 mM PMSF, and 10 mg/ml leupeptin (pH 7.5)). Suspended cells were homogenized mechanically by using a 23-gauge needle and then incubated for 10 min. Cell lysates were centrifugated at 8000 r.p.m. for 5 min at 4 °C. Supernatant, as the non-nuclear fraction, was collected. The obtained pellet, as the nuclear fraction, was then lysed with RIPA lysis buffer containing proteinase and phosphatase inhibitor.

### Isolation of mitochondria

Samples were extracted using the commercial mitochondria isolation kit for cultured cells (Thermo Fisher) according to the manufacturer's manual. Collected cells were suspended in Reagent A, and then incubated for 2 min on ice. Reagent B was added to samples, and then incubated for 5 min. Subsequently, Reagent C was added to samples. Cell lysates were centrifugated at 3000 g for 15 min. Supernatant, as cytosol fraction, was collected. The obtained pellet, as mitochondrial fraction, was then lysed with 2% CHAPS in Tris-buffered saline (25 mM Tris, 0.15 M NaCl; pH 7.2).

### Immunoprecipitation

Collected cells were lysed with non-denaturing lysis buffer (20 mM Tris-HCl pH8, 136 mM NaCl, 1% NP40, and 2 mM EDTA) including proteinase and phosphatase inhibitor. Immunoprecipitation lysates were incubated with an appropriate primary antibody overnight. Subsequently, Protein A/G PLUS-Agarose immunoprecipitation reagents (Santa Cruz Biotechnology) were added to lysates for 4 h. Samples were washed twice with non-denaturing lysis buffer. Similar amounts of samples were loaded in 10% SDS-polyacryl-amide gel for electrophoresis and then transferred to a PVDF membrane. Blotting bands were visualized with an enhanced chemiluminescence solution (Bio-Rad).

### mRNA isolation and reverse transcription polymerase chain reaction

The mESC RNA was extracted by using the RNeasy Plus Mini Kit (QIAGEN, Valencia, CA, USA). Extracted RNA (1 *μ*g) was reverse-transcripted with a Maxime RT premix kit (iNtRON Biotechnology, Sungnam, Korea). The cDNA was amplified with the sense and antisense primers of *gpat1* and *β-actin*.

### Real-Time PCR

Expressions of the *fasn, acc1, cpt1a, cpt1b, magl, gpat1, gpat2, gpat3, gpat4, scd1, scd2, scd3, scd4, lpaatα*, lpaatβ, lpaatδ, lpaatɛ, and *β-actin* genes were detected by using a Rotor-Gene 6000 real-time thermal cycling system (Corbett Research, Mortlake, NSW, Australia) with a QuantiMix SYBR kit (Phile Korea Technology, Daejeon, Korea), mRNA primers, and 1 *μ*g of the cDNA sample. Data were analyzed by using the manufacture's software (Corbett Research). Melting curve analysis was used to confirm the identity and specificity of the PCR products. Normalization was performed by using *β*-actin as the endogenous control. Primer sequences used in experiments are described in Supplementary Table S1.

### Small interfering RNA transfection

Prior to transplantation or hypoxia treatment, siRNAs specific for *gpat1* or NT siRNA as a negative control were transfected to mESCs for 24 h with HiPerfect transfection reagent (QIAGEN) according to the manufacturer's description. Each transfected siRNA's concentration was 25 nM. The siRNA sequences are described in Supplementary Table S2.

### Trypan blue exclusion cell viability assay

Cell-cultured medium was collected and the cells were washed twice with ice-cold PBS. Washed cells were suspended with a 0.05% Trypsin and 0.5 mM EDTA solution. The collected medium was added to the cell suspension solution, including detached cells, and the mixture was treated with soybean trypsin inhibitor (0.05 mg/ml) to quench trypsin activity. Trypan blue (0.4% Sigma-Aldrich) was added to the cell suspension to stain the dead cells. Stained cells and all cells were counted by using a Petroff–Hausser counting chamber (Hausser Scientific, Horsahm, PA, USA). Cell viability was determined by using the following formula. Cell viability=[1−(number of trypan blue-stained cells**/**number of total cells) × 100].

### Annexin V/PI Fluorescence-activated cell sorter analysis

Annexin and PI staining was performed by using Annexin V and PI staining kit (BD Bioscience) according to the manufacturer's instruction. Briefly, cell-cultured medium was collected. The mESCs were detached with a 0.05% Trypsin and 0.5 mM EDTA solution and the collected medium was added to the cell suspension, followed by the addition of soybean trypsin inhibitor (0.05 mg/ml). Next, 1 × 10^5^ cells were resuspended in 1 × binding buffer, immunostained with 5 *μ*l of FITC Annexin V and 5 *μ*l of PI, and incubated at 25 °C for 15 min in the dark. Analysis was performed by using flow cytometry (Beckman Coulter, Fullerton, CA, USA). From each sample, 5 × 10^3^ cells were obtained and analyzed by using CXP software (Beckman Coulter).

### Intracellular reactive oxygen species (ROS) detection

Intracellular ROS was detected by using CM-H_2_DCF-DA (DCF-DA, Life Technologies, Gaithersburg, MD, USA), a H_2_O_2_-sensitive fluorophore. Cells were detached with a 0.05% Trypsin and 0.5 mM EDTA solution, and 5 × 10^5^ cells were resuspended in 10 *μ*M DCF-DA in PBS and incubated at 25 °C for 30 min in the dark. In order to quantify the intracellular ROS levels, the cells were rinsed twice with ice-cold PBS followed by resuspension in ice-cold PBS. A 150 *μ*l aliquot of cell suspension was loaded into a 96-well plate and fluorescence was detected by using a luminometer (Victor3, Perkin-Elmer, Waltham, MA, USA) with excitation and emission wavelengths of 485 and 535 nm, respectively.

### Immunofluorescence staining

Cells were fixed with 80% acetone in PBS for 10 min, followed by washing with PBS. Sequentially, cells were incubated with 5% normal goat serum to inhibit non-specific binding of antibody, and then incubated with primary antibody (1:100 dilution) overnight at 4 °C. Next, the cells were incubated for 1 h with Alexa flour 488-conjugated anti-rabbit and anti-mouse IgG secondary antibody (1:100 dilution, Life Technologies) and PI in PBS. Images were acquired via a FluoView 300 fluorescence microscope (Olympus). For quantification of protein expression, we calculated the corrected total cell fluorescence (CTCF, ImageJ arbitrary unit) by using ImageJ software. The equation used to determine CTCF was CTCF=Integrated density—(Area of selected cell × Mean fluorescence of background readings). Three independent experiments were performed, and the CTCF of 15 colonies (5 colonies per experiment) were obtained.

### Live-cell imaging

After glucosamine or LPA treatment, the culture dish was placed in a temperature and CO_2_ control chamber (Tokai, Tokyo, Japan). Normoxic or hypoxic gas was supplied for 48 h. Differential interference contrast images were acquired over those 48 h at 10 min intervals by using an Olympus IX81-ZDC zero-drift microscope and a Cascade 512B camera (Roper Scientific, Tucson, AZ, USA). A constant threshold of each image was maintained. Determination of the mESC colony area was performed by using ImageJ software.

### [^3^H]-Thymidine incorporation

After treatment, the mESCs were treated with 1 *μ*Ci of [methyl-^3^H]-thymidine (Amersham Biosciences, Piscataway, NJ, USA), and incubated for 1 h at 37 °C. The mESCs were washed with cold PBS, fixed in 10% trichloroacetic acid for 1 h, and then washed with cold PBS. The acid-insoluble substance was lysed with 2N NaOH for 4 h. The level of [^3^H]-thymidine incorporation with DNA was detected using a liquid scintillation counter. Data were normalized from absolute counts to controls.

### Statistical analysis

All experimental data are summarized as a mean±standard error. Differences among experimental groups were tested by using ANOVA. To compare some treatment group means with either control or hypoxia treatment means, the Bonferroni–Dunn test was performed. A test result with a *P*-value<0.05 was considered significant.

## Abbreviations

ACC1: acetyl-coenzyme A carboxylase 1
BrdU: bromodeoxyuridine
CPT1: carnitine palmitoyltransferase
ESC: embryonic stem cell
FASN: fatty acid synthase
GlcN: glucosamine
GPAT1: glycerol-3-phophate acyltransferase-1
HBP: hexosamine biosynthetic pathway
hMSCs: human mesenchymal stem cells
HRP: horseradish peroxidase
LPA: lysophosphatidic acid
LPAAT: lysophosphatidic acid acyltransferase
MAGL: monoacylglycerol lipase
mESC: mouse embryonic stem cell
mTOR: mammalian target of rapamycin
N.S: not statistically significant
NT: non-targeting
GlcNAc: O-linked *β*-N-acetyl glucosaminylation
GlcNAc: N-acetyl D-glucosamine
OGT: O-linked N-acetyl glucosamine transferase
PI: propidium iodide
Ptx: pertussis toxin
PVDF: polyvinylidene fluoride
SCD1: stearoyl-CoA desaturase 1
siRNA: small interfering RNA
ROS: reactive oxygen species
