# Supplementary material for: Glycerol-3-phosphate acyltransferase-1 upregulation by O-GlcNAcylation of Sp1 protects against hypoxia-induced mouse embryonic stem cell apoptosis via mTOR activation
Source: Cell Death Dis. 2016 Mar 24;7(3):e2158–. doi: 10.1038/cddis.2015.410 (PMC4823928; doi:10.1038/cddis.2015.410)
Supplement: Supplementary Table S1 [file cddis2015410x1.docx]

**Supplemental Table S1. Sequences of primers used for RT-PCR and real-time PCR**

| Gene | Identification | Sequence (5'-3') | Size (bp) |
| --- | --- | --- | --- |
| *fasn* | Sense | GATGCCATTCCAGGTAAATG | 147 |
|  | Antisense | CCAGAGGAAGTCAGATGATAG |  |
| *cpt1a* | Sense | TGTGGACCTGCATTCCTTCC | 301 |
|  | Antisense | CAGGTGCTGGTGCTTTTCAC |  |
| *cpt1b* | Sense | CGAGAACTCGTACCAAGTA | 301 |
|  | Antisense | TTGGAGGTCTTGTTTCTTATG |  |
| *magl* | Sense | GCAGAGTGAGGGAGAGAGGA | 293 |
|  | Antisense | TGCGCCCCAAGGTCATATTT |  |
| *gpat1* | Sense | GGTTTGGGACTTGCACGTTC | 327 |
|  | Antisense | GTGCCTTGTGTGCGTTTCAT |  |
| *gpat2* | Sense | GCTAGGTGAATGTAGTGTTG | 329 |
|  | Antisense | TCAATAGGCTCAGTGTATGT |  |
| *gpat3* | Sense | AGGTTCTCCTCCGAAGAGCT | 352 |
|  | Antisense | GAGAGGTGTGATTGGCGACA |  |
| *gpat4* | Sense | GCCCTACACCAACGGAATCA | 197 |
|  | Antisense | GCTCCTCTGCCGAGAATCTC |  |
| *scd1* | Sense | TTGGCTAGCTATCTCTGCGC | 289 |
|  | Antisense | GGCCGGCATGATGATAGTCA |  |
| *scd2* | Sense | CTTTCAACATAGCGCGCCTC | 263 |
|  | Antisense | GCCTCTCCAGTCCTGCAATT |  |
| *scd3* | Sense | GAAGCACCCATGTTGTATAG | 283 |
|  | Antisense | GAAGCACCCATGTTGTATAG |  |
| *scd4* | Sense | TACCGCTGGCACATCAACTT | 277 |
|  | Antisense | AGACCATGTACCCTCCCCTC |  |
| *lpaat-α* | Sense | CTCGACCTGCTTGGAATG | 237 |
|  | Antisense | GGAGCCATTGTGGTTTCT |  |
| *lpaat-β* | Sense | CATGATGGGTCTCATGGAAA | 325 |
|  | Antisense | GTAGAAGGAAGAGAAAGACGAG |  |
| *lpaat-δ* | Sense | ATGCCTTTCAGGAGGAATAC | 206 |
|  | Antisense | CATGGAGGCCATACAGAAG |  |
| *lpaat-ε* | Sense | CACCATCCATGACTGAGTTT | 197 |
|  | Antisense | CCCAGGAAATTTGTTTCTTCTT |  |
| *β-actin* | Sense | GCAGGAGTACGATGAGTCCG | 239 |
|  | Antisense | ATCCTGAGTCAAAAGCGCCA |  |
