# Supplementary material for: Glycerol-3-phosphate acyltransferase-1 upregulation by O-GlcNAcylation of Sp1 protects against hypoxia-induced mouse embryonic stem cell apoptosis via mTOR activation
Source: Cell Death Dis. 2016 Mar 24;7(3):e2158–. doi: 10.1038/cddis.2015.410 (PMC4823928; doi:10.1038/cddis.2015.410)
Supplement: Supplementary Table S2 [file cddis2015410x2.docx]

**Suppplemental Table S2. Sequences of siRNAs used for gene silencing**

| Target gene | Sequence 5'-3' |
| --- | --- |
| *gpat1* | CCACAUACUGUCUCGUGAA |
|  | GGUGAGGAGCAGCGAGAUU |
|  | CGUACAUCGCCUCGGGCAA |
|  | CUUAUCACCAGGACGGAAA |
| Non-targeting | UAGCGACUAAACACAUCAA |
|  | UAAGGCUAUGAAGAGAUAC |
|  | AUGUAUUGGCCUGUAUUAG |
|  | AUGAACGUGAAUUGCUCAA |
