# Supplementary figures and images for: Glycerol-3-phosphate acyltransferase-1 upregulation by O-GlcNAcylation of Sp1 protects against hypoxia-induced mouse embryonic stem cell apoptosis via mTOR activation
Source: Cell Death Dis. 2016 Mar 24;7(3):e2158–. doi: 10.1038/cddis.2015.410 (PMC4823928; doi:10.1038/cddis.2015.410)

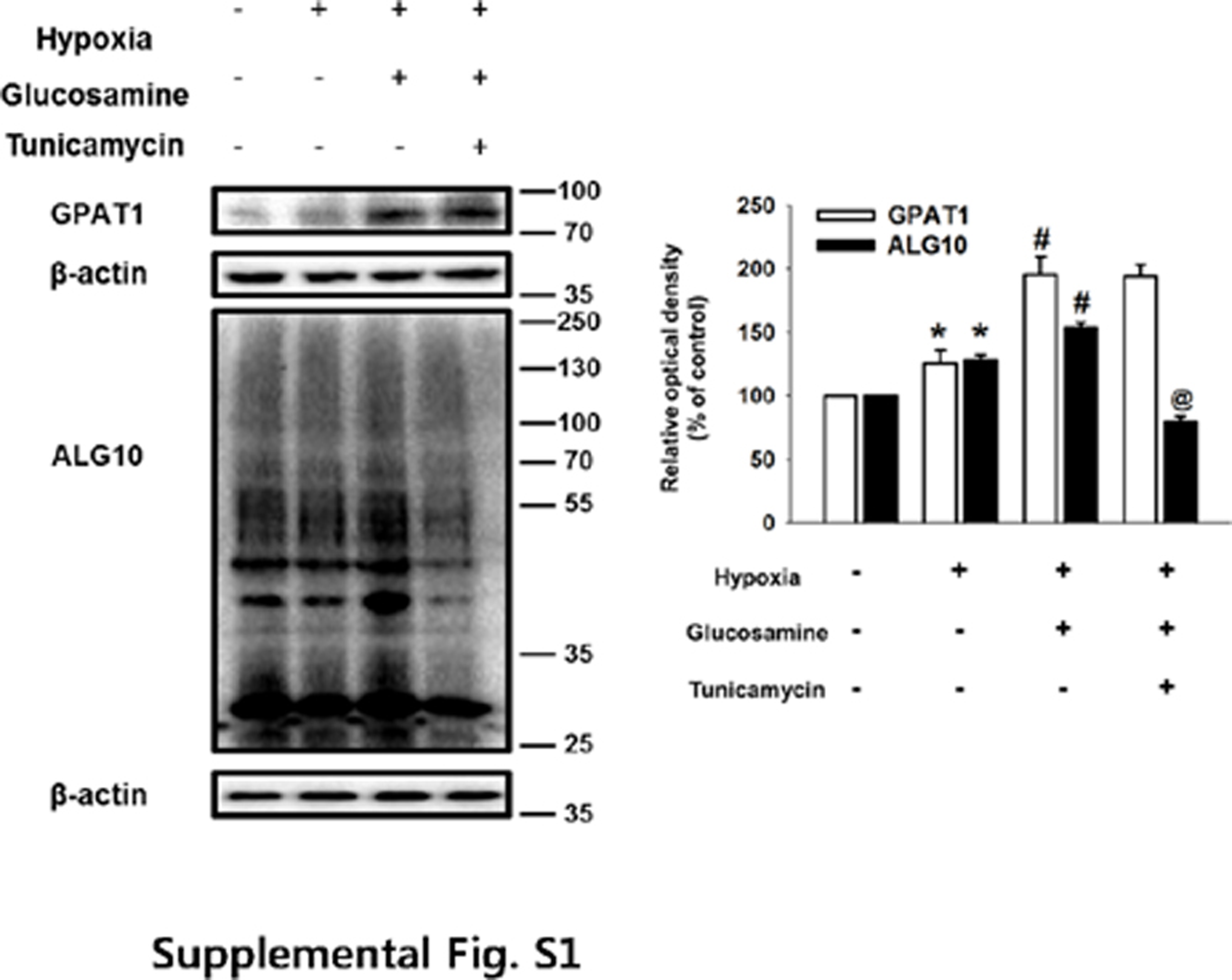

Supplement: Supplementary Figure S1 [file cddis2015410x3.tif]

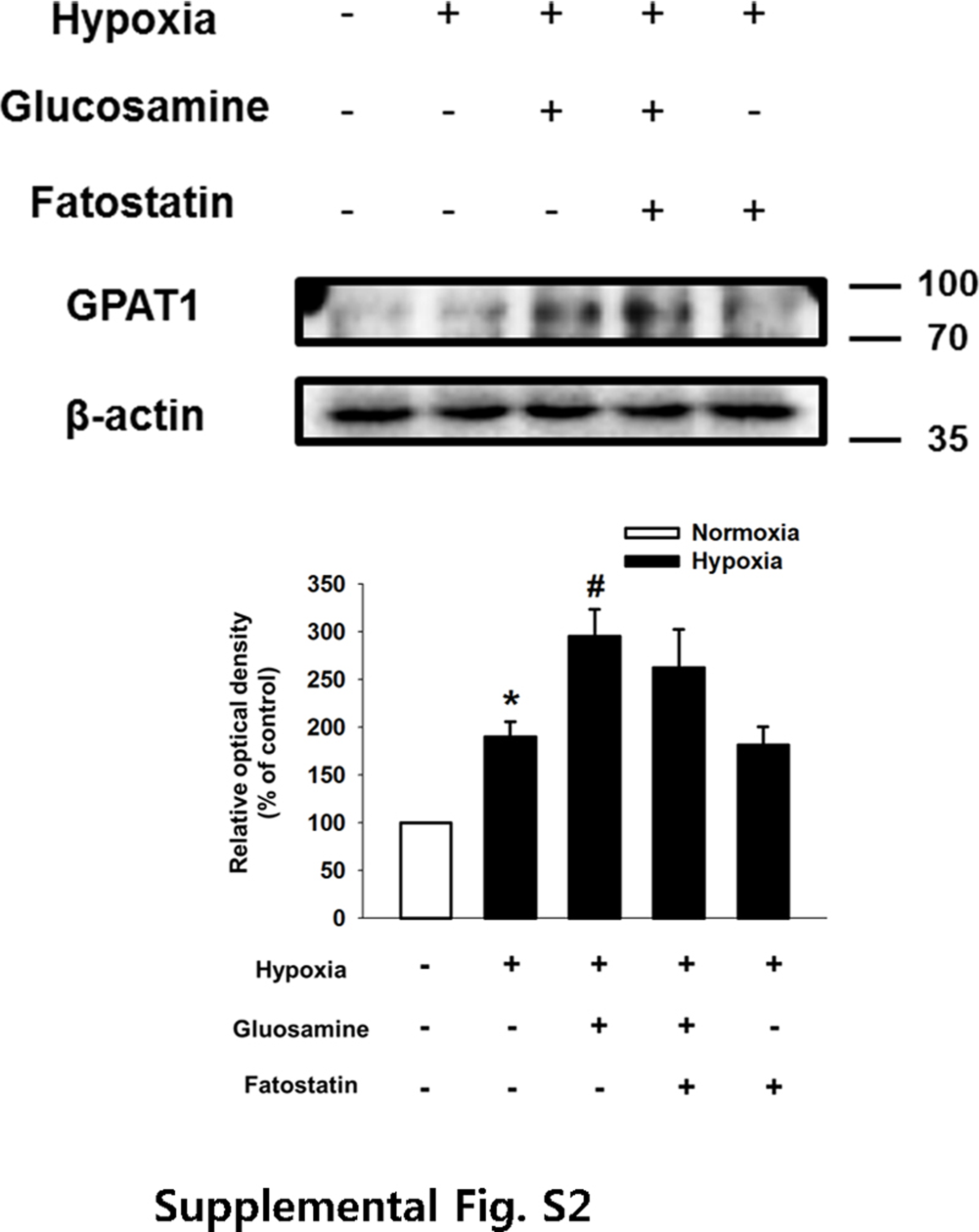

Supplement: Supplementary Figure S2 [file cddis2015410x4.tif]

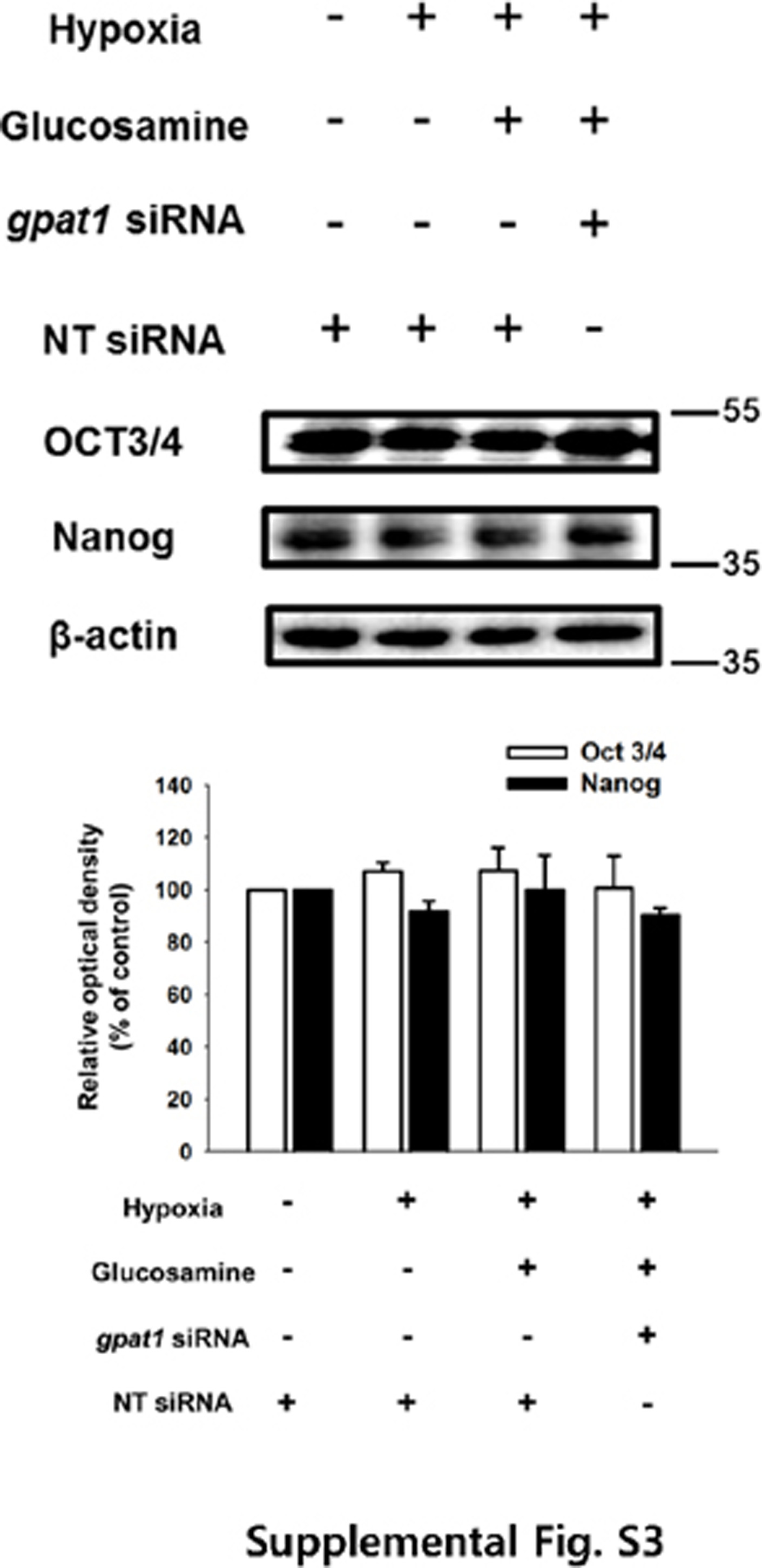

Supplement: Supplementary Figure S3 [file cddis2015410x5.tif]

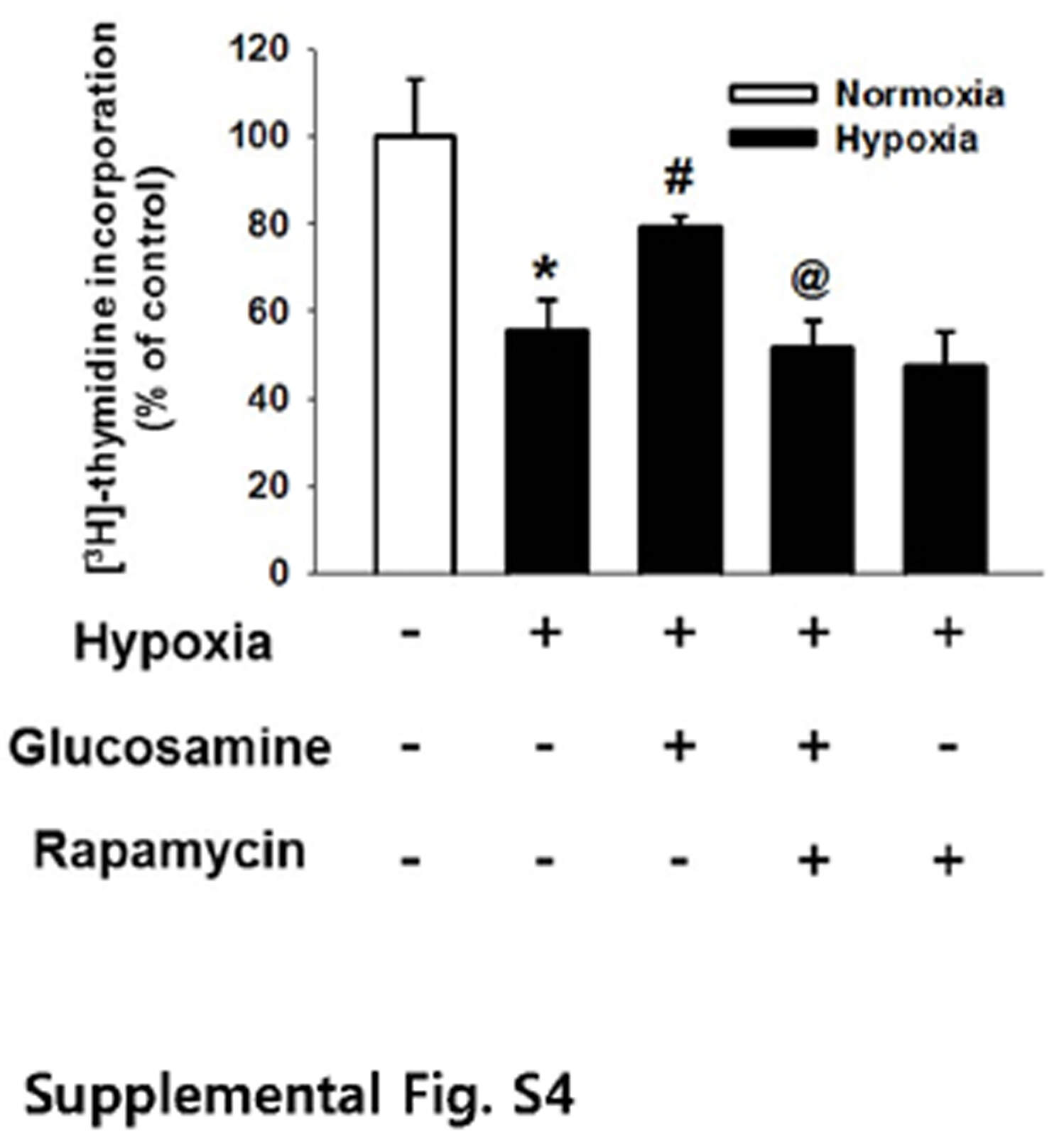

Supplement: Supplementary Figure S4 [file cddis2015410x6.tif]

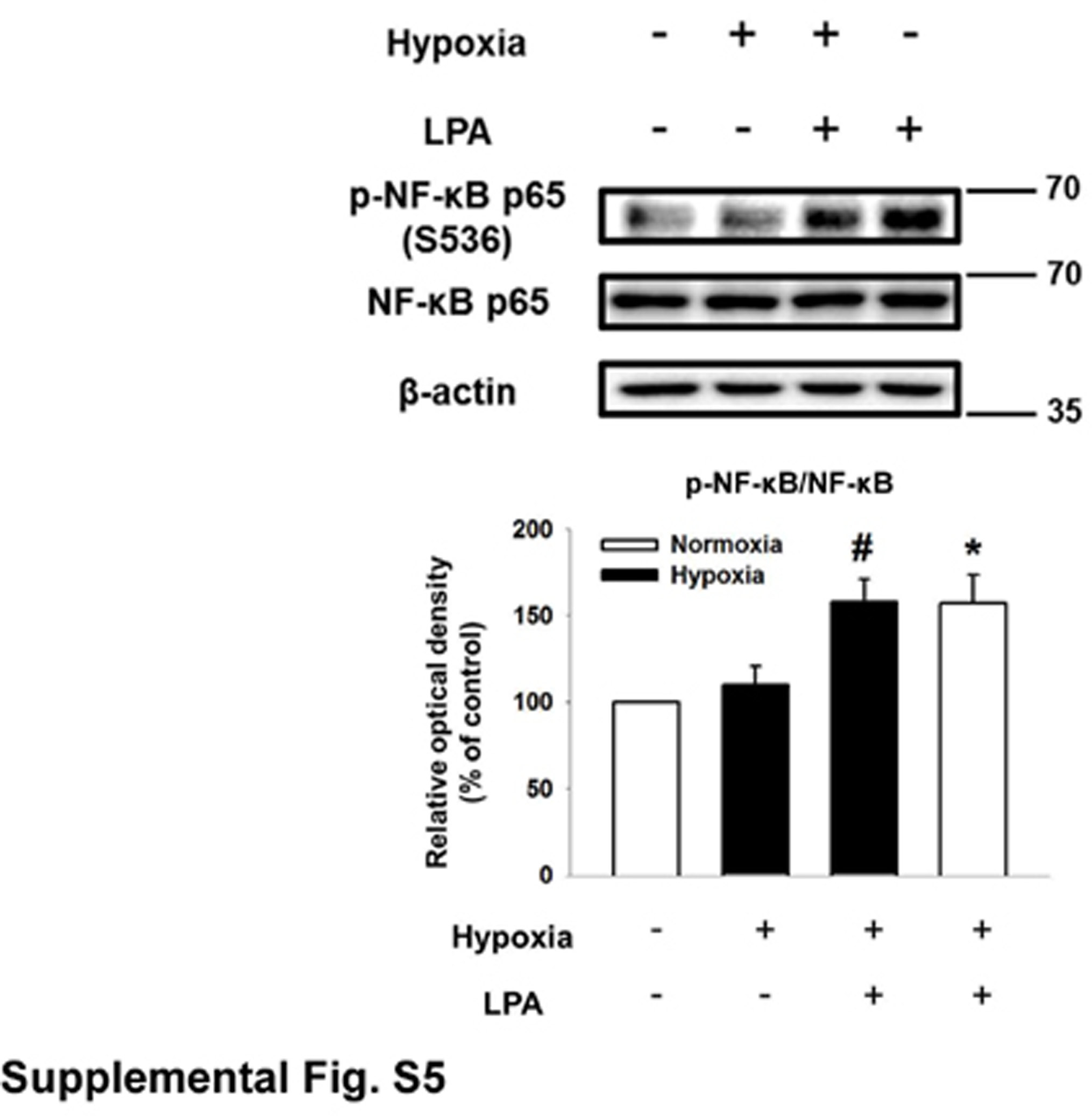

Supplement: Supplementary Figure S5 [file cddis2015410x7.tif]

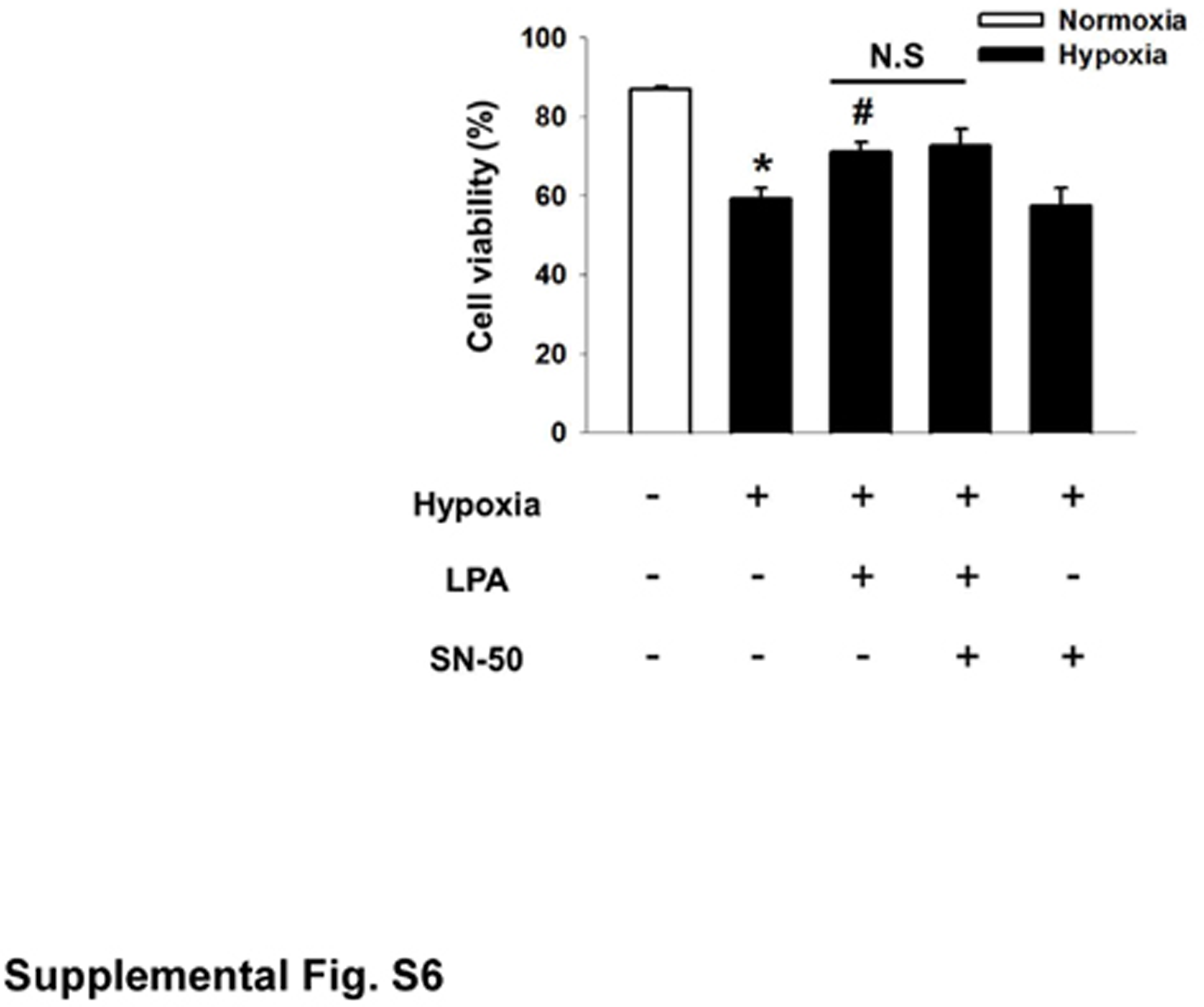

Supplement: Supplementary Figure S6 [file cddis2015410x8.tif]
