# Supplementary material for: Glycerol-3-phosphate acyltransferase-1 upregulation by O-GlcNAcylation of Sp1 protects against hypoxia-induced mouse embryonic stem cell apoptosis via mTOR activation
Source: Cell Death Dis. 2016 Mar 24;7(3):e2158–. doi: 10.1038/cddis.2015.410 (PMC4823928; doi:10.1038/cddis.2015.410)
Supplement: Supplementary Information [file cddis2015410x12.doc]

Supplemental figure S1. Role of N-linked glycosylation in glucosamine-induced GPAT1 expression. Cells were pretreated with N-linked glycosylation inhibitor tunicamycin (0.1 μg/ml) prior glucosamine (10 μM) treatment. Subsequently, cells were treated with hypoxia for 24 h. Total N-linked glycosylation was blotted by using RL-2 N-linked glycosylation specific antibody. GPAT1, ALG10, and β-actin were detected by using western blotting. Each result shown is representative of three independent experiments. * indicates *p* < .05 versus control, # indicates *p* < .05 versus hypoxia treatment, and @ indicates *p* < .05 versus hypoxia with glucosamine.

Supplemental figure S2. Role of SREBP1 in glucosamine induced GPAT1 expression under hypoxia. Cells were pretreated with fatostatin (1 μM) prior to glucosamine (10 μM) treatment. And then cells were exposed to hypoxia for 24 h. Total proteins were extracted, and blotted with GPAT1 and β-actin specific antibodies. Each image data is representative of three independent experiments. * indicates *p* < .05 versus control, # indicates *p* < .05 versus hypoxia treatment, and @ indicates *p* < .05 versus hypoxia with glucosamine.

Supplemental figure S3. Role of glucosamine-induced GPAT1 in undifferentiation markers expression under hypoxia. Cells were transfected with *gpat1* and non-targeting siRNA. And then, cells were exposed to hypoxia for 24 h. Total proteins were extracted, and blotted with Oct3/4, nanog and β-actin specific antibodies. Each image is representative of three independent experiments.

Supplemental figure S4. Role of glucosamine and mTOR on [^3^H]-thymidine incorporation of mESCs under hypoxia. Cells were pretreated with rapamycin (10 nM) prior glucosamine treatment. Subsequently, cells were exposed to hypoxia for 24 h, and then pulsed with 1 μCi of [^3^H]-thymidine for 1 h prior to counting. Data are reported as a mean ± SE. n=6. * indicates *p* < .05 versus control, # indicates *p* < .05 versus hypoxia treatment, and @ indicates *p* < .05 versus hypoxia with glucosamine.

Supplemental figure S5. Role of LPA in NF-κB phosphorylation on mESCs under hypoxia. The mESCs were treated with hypoxia or LPA. And then, p-NF-κB p65 (S536), NF-κB p65 and β-actin were detected by using western blotting. The bottom panel depicted by bars denoted mean ± S.E. Each result shown is representative of three independent experiments. * indicates *p* < .05 versus control, and # indicates *p* < .05 versus hypoxia treatment alone.

Supplemental figure S6. Role of LPA-induced NF-κB in mESC survival under hypoxia. Measurement of cell viability was performed by trypan blue exclusion cell viability assay. Cells were pretreated with SN-50 (1 μM) prior to LPA (0.1 μM) for 30 min. Subsequently, cells were exposed to 24 h of hypoxia. Data are presented as a mean ± S.E of three independent duplex dishes. * indicates *p* < .05 versus control, and # indicates *p* < .05 versus hypoxia treatment alone. N.S indicates not statistically significant.

Supplementary information is available at Cell Death & Disease’s website.
